# Climate Cycles and Forecasts of Cutaneous Leishmaniasis, a Nonstationary Vector-Borne Disease

**DOI:** 10.1371/journal.pmed.0030295

**Published:** 2006-08-15

**Authors:** Luis Fernando Chaves, Mercedes Pascual

**Affiliations:** Department of Ecology and Evolutionary Biology, University of Michigan, Ann Arbor, Michigan, United States of America; University of Wisconsin, United States of America

## Abstract

**Background:**

Cutaneous leishmaniasis (CL) is one of the main emergent diseases in the Americas. As in other vector-transmitted diseases, its transmission is sensitive to the physical environment, but no study has addressed the nonstationary nature of such relationships or the interannual patterns of cycling of the disease.

**Methods and Findings:**

We studied monthly data, spanning from 1991 to 2001, of CL incidence in Costa Rica using several approaches for nonstationary time series analysis in order to ensure robustness in the description of CL's cycles. Interannual cycles of the disease and the association of these cycles to climate variables were described using frequency and time-frequency techniques for time series analysis. We fitted linear models to the data using climatic predictors, and tested forecasting accuracy for several intervals of time. Forecasts were evaluated using “out of fit” data (i.e., data not used to fit the models). We showed that CL has cycles of approximately 3 y that are coherent with those of temperature and El Niño Southern Oscillation indices (Sea Surface Temperature 4 and Multivariate ENSO Index).

**Conclusions:**

Linear models using temperature and MEI can predict satisfactorily CL incidence dynamics up to 12 mo ahead, with an accuracy that varies from 72% to 77% depending on prediction time. They clearly outperform simpler models with no climate predictors, a finding that further supports a dynamical link between the disease and climate.

## Abstract 2

Using mathematical models, the authors show that cutaneous leishmaniasis has cycles of approximately three years that are related to temperature cycles and indices of the El Niño Southern Oscillation.

## Editors' Summary

**Background.**

Every year, 2 million people become infected with a pathogenic species of *Leishmania,* a parasite that is transmitted to humans through the bites of infected sand flies. These flies—the insect vectors for disease transmission—pick up parasites by biting infected animals—the reservoirs for the parasite. Once in a person, some species of *Leishmania* can cause cutaneous leishmaniasis, a condition characterized by numerous skin lesions. These usually heal spontaneously but can leave ugly, sometimes disabling scars. Leishmaniasis is endemic and constantly present in many tropical and temperate countries, but as with other diseases that are transmitted by insect vectors (for example, malaria), the occurrence of cases has a strong seasonal pattern and also varies from year to year (interannual variability). These fluctuations suggest that leishmaniasis transmission is sensitive to seasonal changes in the climate and to climatic events like the El Niño Southern Oscillation (ENSO), a major cause of interannual weather and climate variation around the world that repeats every 3–4 years. This sensitivity arises because the climate directly affects the abundance of sand flies and how quickly the parasites replicate.

**Why Was This Study Done?**

It would be very useful to have early warning systems for leishmaniasis and other vector-transmitted diseases so that public health officials could prepare for epidemics—or spikes in the number of cases—of these diseases. Monitoring of climatic changes could form the basis of such systems. But although it is clear that the transmission of cutaneous leishmaniasis is affected by fluctuations in the climate, there have been no detailed studies into the dynamics of its seasonal or yearly variation. In this study, the researchers used climatic data and information about cutaneous leishmaniasis in Costa Rica to build statistical models that investigate how climate affects leishmaniasis transmission.

**What Did the Researchers Do and Find?**

The researchers obtained the monthly records for cutaneous leishmaniasis in Costa Rica for 1991 to 2001. They then used several advanced statistical models to investigate how these data relate to climatic variables such as the sea surface temperature (a measure of El Niño, a large-scale warming of the sea), average temperature in Costa Rica, and the MEI (the Multivariate ENSO Index, a collection of temperature and air pressure measurements that predicts when the ENSO is going to occur). Their analyses show that cutaneous leishmaniasis cases usually peak in May and that the incidence of the disease (number of cases occurring in the population over a set time period) rises and falls in three-year cycles. These cycles, they report, match up with similar-length cycles in the climatic variables that they investigated. Furthermore, when the researchers tested the models they had constructed with data that had not been used to construct the models (“out of fit” data), the models predicted variations in the incidence of cutaneous leishmaniasis for up 12 months with an accuracy of about 75% (that is, the predictions were correct three times out of four).

**What Do These Findings Mean?**

The finding that interannual cycles of climate variables and cutaneous leishmaniasis coincide provides strong evidence that climate does indeed affect the transmission of this disease. This link is strengthened by the ability of the statistical models described by the researchers to predict outbreaks with high accuracy. The researchers' new insights into how climate affects the transmission of cutaneous leishmaniasis are important because they open the door to the possibility of setting up an early warning system for this increasingly common disease. The same statistical approach could be used to improve understanding of how climate affects the dynamics of other vector-transmitted diseases and to design early warning systems for them as well—the World Health Organization has identified 18 diseases for which climate-based early warning systems might be useful but no such systems are currently being used to plan disease control strategies. Finally, the improved understanding of the relationship between climate and disease transmission that the researchers have gained through their study is an important step towards being able to predict how the incidence and distribution of leishmaniasis and other vector-transmitted diseases will be affected by global warming.

**Additional Information.**

Please access these Web sites via the online version of this summary at http://dx.doi.org/10.1371/journal.pmed.0030295.

- United States Centers for Disease Control and Prevention fact sheet on leishmaniasis
- MedlinePlus encyclopedia entry on leishmaniasis
- World Health Organization information on leishmaniasis and on climate change and health
- Wikipedia pages on leishmaniasis and on the El Niño Southern Oscillation (note that Wikipedia is a free online encyclopedia that anyone can edit)

## Introduction

The leishmaniases are among the most important emerging and resurging vector-borne protozoal diseases, second only to malaria in terms of the number of affected people. Like malaria, the leishmaniases can be caused by infection with any of several species of parasites belonging to the genus *Leishmania* (Kinetoplastida: Trypanosomatidae) and transmitted by sand flies (Diptera: Psychodidae) [1–3]. The disease has two main clinical manifestations: visceral and cutaneous/mucocutaneous. The latter is caused by at least 14 different species of parasites belonging to the subgenera *Viannia* and *Leishmania* [4]. However, it has been suggested that the cutaneous manifestation can be due to heterogeneities in the hosts [5]. An interesting aspect of the dynamics of this disease is that it is strongly associated with the presence of reservoirs, animals that act as sources and sinks of infection to sand flies, while humans are considered to be only incidental hosts, i.e., sinks for infections [3,6].

As with other vector-borne diseases, seasonal patterns in cases and vector abundance suggest that cutaneous leishmaniasis (CL) transmission is sensitive to climatic exogenous factors. More specifically, vector density is correlated to climate variables—producing seasonal patterns that have been widely described [7–10]—vector density is correlated with number of cases [9,11], transmission is restricted to wet and forested areas [12], and vector density diminishes with altitude [13]. Also, interannual climatic events related to the El Niño Southern Oscillation (ENSO) have been shown to be associated with outbreaks of visceral leishmaniasis at the annual level [14]. However, the interannual cycles of CL have not been examined, and disease data have not been considered at the higher temporal resolution of months, more relevant to the seasonal dynamics of transmission. While many studies of climate–disease couplings have emphasized the potential application of associations between climate and disease to early warning systems, the forecasting ability of the resulting models has not been systematically evaluated. This critical step should be carried out with “out of fit” data if such models are to become useful tools to guide public health policy [3]. In addition, analyses of climate–disease relationships must take into account a common property of disease data that can mask the patterns: time series of cases are typically nonstationary, with changes in the mean and/or the variance over time.

In the present paper, CL cycles and their relationship to climate variables are described, and linear statistical models that use climate variables as predictors are used to assess the accuracy of forecasts based on climatic variables.

## Methods

### Data

**Figure 1:**
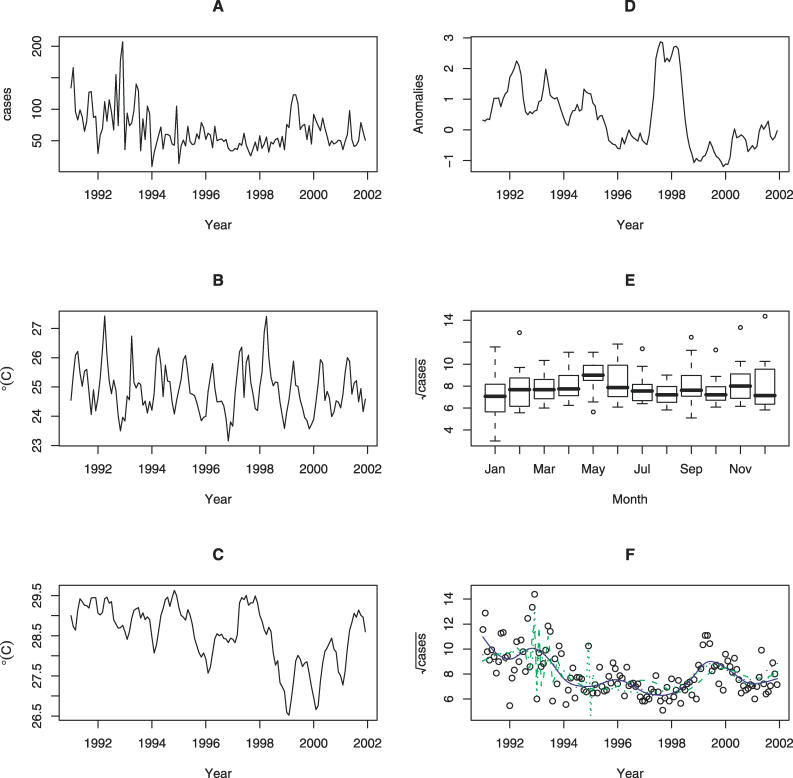
Time Series (A) CL cases in Costa Rica. (B) Mean temperature in Costa Rica. (C) SST 4. (D) MEI. (E) Box plot with monthly square-root-transformed CL cases. (F) The fits of (1) the Daubechies discrete wavelet (green lines), used to detrend the series so that the resulting data can then be analyzed for their dominant frequencies with a periodogram (a filter number 5 and eight levels of decomposition were used for this wavelet; the dashed line corresponds to periodic edges, and the dotted line to symmetric ones); (2) smoothing splines (blue solid line) and the first four reconstructed components of singular spectrum analysis (black dashed line; 60 orders). These methods were used to de-noise the signals so that dominant frequencies could be identified (with the maximum entropy spectral density method).

Monthly records of CL cases, from January 1991 to December 2001, were obtained from the epidemic surveillance service, Vigilancia de la Salud, of Costa Rica. Data were normalized using a square root transformation. Sea Surface Temperature 4 (SST 4) (also known as the Niño 4 index; http://www.cpc.ncep.noaa.gov/data/indices) and the average temperature of the 0.5° × 0.5° grids corresponding to the Costa Rica land surface (http://www.cru.uea.ac.uk; [15]) were used as climatic variables to study the patterns of association between climate and CL. Temperature, SST 4, and the Multivariate ENSO Index (MEI) (http://www.cdc.noaa.gov/people/klaus.wolter/MEI; [16]) were used as predictors for forecasting CL cases. All the time series are presented in Figure 1.

### Statistical Analysis

#### Seasonality.

The seasonality of CL cases was assessed by using a box diagram (see Figure 1E) [17].

#### Interannual cycles of CL.

A time series is stationary if it has a constant mean and variance [17–19]. Therefore, a nonstationary time series is one whose mean and/or variance is nonconstant. As Figure 1A shows, CL is nonstationary as it has a changing mean, an observation that can be confirmed by inspecting its autocorrelation function (see Figure S1). Because the disease is nonstationary, we used several methods of analysis to obtain robust results about the characteristic temporal scales of the cycles. The cycling patterns of a time series, *y_t_,* can be studied in the frequency domain and the time-frequency domain [18]. In the frequency domain, we used two main general approaches to determine the dominant frequencies in the data. The first one consisted of computing the periodogram, which gives the distribution of power (or, equivalently, variance) among different frequencies (see [19] and Protocol S1 for details). Thus, a peak in the periodogram indicates a dominant frequency. However, the periodogram assumes that the time series is stationary (i.e., with a constant mean and variance). Because our data are clearly nonstationary, we detrended the time series using the method known as discrete wavelet shrinkage (described in detail in Protocol S1).

The second method consisted of the maximun entropy spectral density *Y*(*v_k_*) and was computed using the parameters 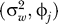 of a *p*th order autoregressive process fitted to the data (see [20] and Protocol S1 for details).

The above characterizations of the cycles consider the whole temporal extent of the data and therefore provide, as such, only an average picture of dominant frequencies in the data. More recently, the importance of localizing these frequencies in time has been emphasized, particularly for nonstationary data. The wavelet power spectrum (WPS*_y_*) allows us to do so by calculating a measure of power as a function of both frequency and time. In other words, we can determine when in the temporal record a particular frequency is dominant and significant ([21,22]; see Protocol S1 for technical details).

#### Patterns of association between climate variables and CL.

Besides allowing us to determine how the variability of the data is allocated to different frequencies at different times, the wavelet transform can be used to study the patterns of association between two nonstationary time series [21]. Specifically, with the wavelet coherency analysis, we can determine whether the presence of a particular frequency at a given time in the disease corresponds to the presence of that same frequency at the same time in a climate covariate, and with the cross-wavelet phase analysis we can determine the time lag separating these two series as well.

####  Linear models and forecasts.

Seasonal autoregressive models were fitted to the data using the Kalman recursions for their state space representation ([23]; see Protocol S1 for technical details). To select the lags for the climate variables the following procedure was used [19,23,24]: (i) a null model was fitted to the square-root-transformed cases, (ii) MEI, SST 4, and temperature were filtered with the coefficients of the null model, and (iii) cross-correlation functions were computed using the residuals of the null model and those of the filtered climatic variables.

On the basis of the cross-correlation functions, a full model was fitted that included as predictors all the statistically significant lagged climate variables [19]. This model was then simplified based on the following criteria: (i) the minimization of the Akaike information criterion and (ii) the absence of a significant difference (*p* < 0.05) when comparing the full model with the simpler version through a chi-squared likelihood ratio test [18,19,23].

Forecasts were obtained for time intervals 1, 3, 6, and 12 mo ahead using a total of 24 mo for each time interval. The model was refitted before computing the next prediction in two different ways, by including (i) all the previous months in the series or (ii) only the months from the previous 9 y.

The accuracy of the forecast was measured using the predictive *R* ^2^, which has an interpretation similar to the *R* ^2^ of a linear regression as defined in [25] and is given by (1 – mean squared error/variance of the series). Forecast accuracy was tested for the model selected as best, as well as for the simpler versions of this model, including a null model without climate covariates.

## Results

**Figure 2:**
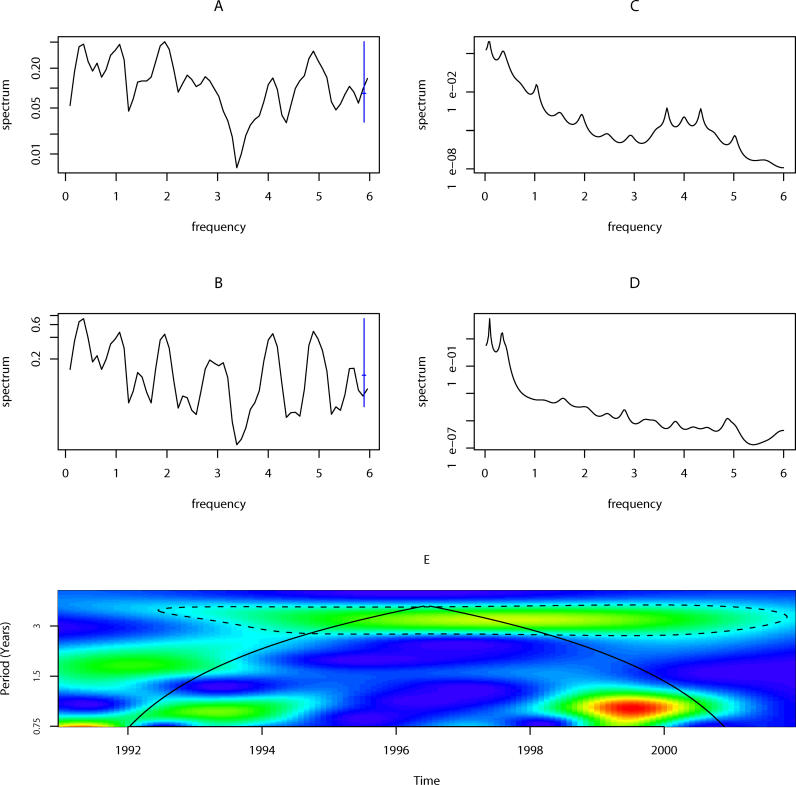
Dominant Frequencies in the Data (A and B) Smoothed periodograms for (A) the detrended series (using Daubechies discrete wavelet with filter number 5 and periodic edges) and (B) the detrended series (using Daubechies discrete wavelet with filter number 5 and symmetric edges). In the periodograms, the blue lines are the 95% point confidence intervals [19]. (C and D) Maximum entropy spectral density for (C) the de-noised series (with smoothing splines) and (D) the de-noised series (with singular spectrum analysis). For the periodograms and the maximum entropy spectral density, frequencies are in cycles per year. (E) Wavelet power spectrum. The solid line is the cone of influence indicating the region of time and frequency where the results are not influenced by the edges of the data and are therefore reliable. The dashed line corresponds to the 95% confidence interval for white noise based on the variance of the square-root-transformed incidence series. The intervals were obtained using a chi-squared distributed statistic with one degree of freedom (see [22] for details). The Morlet wavelet was used [18,21,22]. In all analyses, the cases are square-root-transformed. Maximum entropy spectral densities were computed using the software described in [20]. For the maximum entropy spectral density an autoregressive process of order *p* = 40 was used, i.e., AR(40).

**Figure 3:**
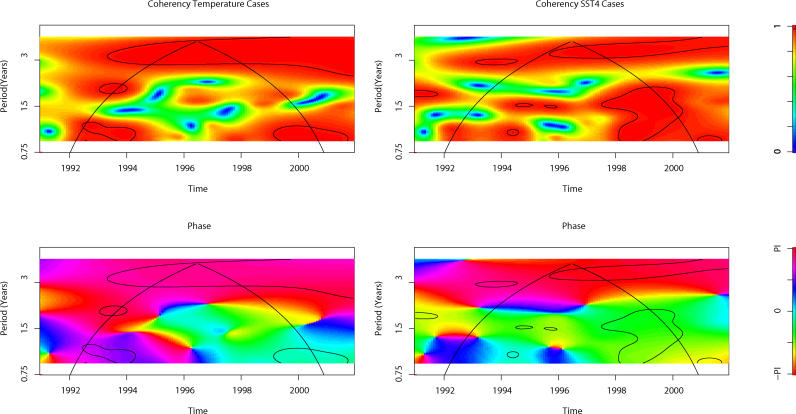
Cross-Wavelet Coherency and Phase The coherency scale is from zero (blue) to one (red). Thus, red regions indicate frequencies and times for which the two series share variability. The cone of influence (within which results are not influenced by the edges of the data) and the significant (*p* < 0.05) coherent time-frequency regions are indicated by solid lines. The colors in the phase plots correspond to different lags between the variability in the two series for a given time and frequency, measured in angles from −PI to PI. A value of PI corresponds to a lag of 16 mo. Cases are square-root-transformed. The procedures and software are those described in [21]. A smoothing window of 15 mo (2w + 1 = 31) was used to compute the cross-wavelet coherence.

**Figure 4:**
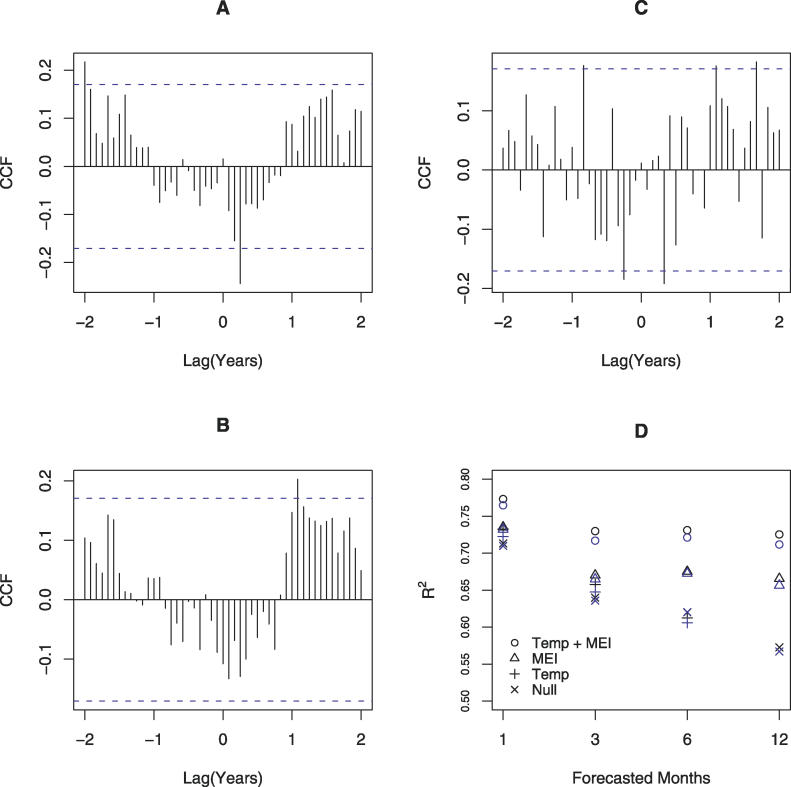
Cross-Correlation Functions of Square-Root-Transformed Cases with SST 4, Temperature, and MEI (A–C) Cross-correlation functions (CCF) with (A) SST 4, (B) MEI, (C) Temperature. The blue dashed lines are the 95% point confidence intervals for the cross-correlation between two series that are white noise [23]. (D) Predictive *R* ^2^ measuring the accuracy of the predictions. Blue is for predictions with only 9 y of training data (used to fit the model) and black for predictions generated with all months preceding the prediction. (The value for 12-mo predictions with temperature is not shown, because it was negative.)

CL shows, on average, a seasonal peak during May, though epidemic outbreaks happen around the year, as demonstrated by the existence of outliers in February, July, September, October, November, and December (Figure 1E). Interannual cycles with a period of approximately 3.2 y were identified by all the methods, with the exception of maximum entropy spectral density (with smoothing splines), which found cycles of 2.7 y (Figure 2). In addition, cycles of 8 y were found with the maximum entropy spectral density regardless of the method used to de-noise the data. However, evidence for the 8-y cycles is weak as they are longer than half the longitude of the series, a period above which the methods become unreliable [20]. The interannual cycles of 3.2 y coincide with those present in temperature and SST 4 for the same time period, suggesting a possible association between climate and transmission. In fact, cross- wavelet coherency analysis reveals that the square-root-transformed cases are significantly (*p* < 0.05) coherent with temperature and SST 4 at periods around 3 y, with SST 4 and temperature leading the disease data, as shown by the cross-wavelet phases (Figure 3). The difference in phase is longer for SST 4 than for temperature. A similar pattern of coherence and phase is also seen for MEI, with MEI and cases coherent at periods of 3 y, and MEI leading the dynamics of the disease with a longer phase than that of temperature (Figure S2). For both series, the cases are also significantly coherent at the seasonal scale, with only temperature leading cases, while SST 4 follows, possibly an artifact due to the seasonality of both series [21]. The results of cross-wavelet coherence and phase are consistent with those of the cross-correlation functions: the dynamics of the square-root-transformed CL cases are led by temperature with a 4-mo lag and by MEI with a 13-mo lag (Figure 4).

The forecasting accuracy is higher for the model selected by the likelihood ratio tests and Akaike information criterion values (Table 1). The best model describes the following process:

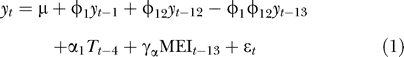
where *μ* is the intercept, 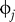 are the autoregressive terms, *α_1_* is a regression parameter for temperature *(T), γ_a_* is a regression parameter for MEI, and the residuals ɛ*_t_* are 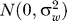 distributed. This model has a predictive accuracy of over 72%. The model with MEI as a predictor outperforms the model with just temperature and the null model with no climate covariates (Figure 4). In fact, the MEI model accounts for more than 65% of the variance in the data for prediction times up to 1 y (Figure 4). By contrast, the predictive *R* ^2^ for the temperature model is negative for predictions 12 mo ahead, and the mean squared error is two orders of magnitude higher than the original variance of the series (Figure S3). As expected, for all the evaluated models, forecasting accuracy diminishes with time and is slightly improved when all the previous months are employed for fitting the model (Figure 4).

**Table 1:**
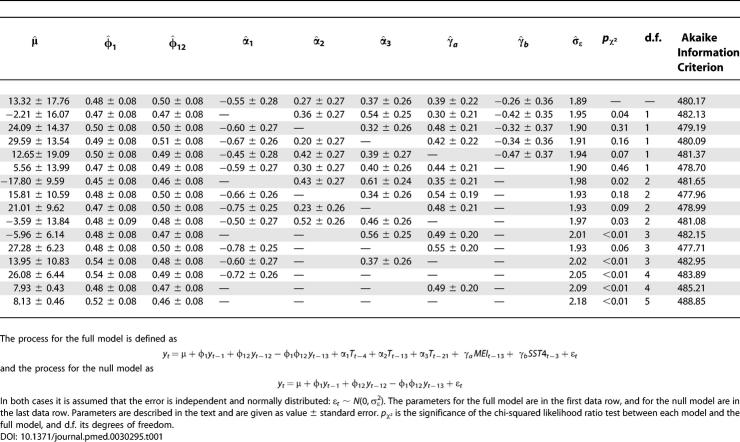
Model Selection and Parameter Values

## Discussion

The description of cycles in any natural phenomenon is relevant to predict its dynamic behavior. The finding of a period close to 3 y for the interannual cycles of CL is robust, as the four applied methods indicate consistently the presence of cycles between 2.7 and 3.2 y in this series. The robustness of this result indicates that its statistical significance is not an artifact of any particular methodology. However, the description of cycles by itself is not sufficient to assess the effects of exogenous drivers or gain insights into the processes driving such oscillations.

A deeper understanding of the relationship between climate and disease dynamics is key for anticipating the potential effects that trends in a changing climate would have on the incidence and distribution of the disease [26]. The results of this study show that the dynamics of CL are strongly associated to those of climate variables, including temperature and ENSO indices, with coherent cycles of around 3 y. Similarly, associations with climate have been found for other vector-transmitted diseases, including dengue [27] and malaria [28–31].

A strong association between climate and CL incidence is further supported here by the finding that linear models can forecast satisfactorily the incidence of this disease, with an accuracy between 72% and 77%. In particular, MEI and temperature are identified as useful variables sustaining predictability for a window of 1 y. Interestingly, MEI is defined as the first principal component of several climate variables that predict ENSO [16]. This type of variable has been known to work well in linear regression because it reduces the number of predictors, avoiding problems of collinearity in the predictor matrix [32,33]. Longer-term data are needed to evaluate forecasting accuracy further in time. In the predictive model the nonstationarity of the CL time series is captured by the climatic covariates and the seasonal autoregressive part of the model.

The finding that the model with MEI as the only predictor outperforms the model with just temperature supports the recent proposal that large-scale climate indices may be more useful for forecasting than local climate variables [32]. Climate can affect through several linear and nonlinear pathways the dynamics of infection in a host population. It can affect several biological traits of the organisms involved in the life cycle of the parasites, from individual life histories to population dynamics [34], and modify several factors that determine the context of disease transmission, including food production and the general standard of living of the population under the changing environment [35], both of which are known as important risk factors for other vector-borne diseases [36]. While local climate is more likely to affect only the biological components of disease transmission, large-scale climate patterns could also influence contextual components of disease dynamics, such as population susceptibility.

For CL there are several plausible ways in which climate could affect transmission dynamics. As already pointed out, vector density is sensitive to climate variability, with vector densities varying seasonally [7–10]. Parasite developmental time in vectors is also sensitive to environmental conditions, decreasing with high temperatures [37]. The density of reservoirs might also be sensitive to climate. For example, hantavirus outbreaks have been associated with changes in rodent reservoir densities, and high densities of rodents correlate with the altered production of seeds as the result of climatic conditions [38,39]. We can also expect there to be contextual effects of climate on transmission, such as those mediated by natural disasters, which could increase the risk of acquiring an infectious disease [35].

Future work should compare the forecasting ability of nonlinear models and more mechanistic formulations. While mechanistic models are necessary to propose and evaluate methods of control [3], there may be trade-offs between the complexity of these models and their ability to predict [3,40]. The results obtained here provide a basis for modeling other aspects of CL [3] and for producing forecasts for windows of time as long as 1 y ahead. The approach described in this paper could be applied to evaluating predictability in other vector-transmitted diseases.

## Supporting Information

Figure S1 The Autocorrelation Function of the Square-Root-Transformed CL Incidence Time Series This series is nonstationary, because the autocorrelation function is statistically significant for lags different from zero and decays over time, a pattern that is superimposed on that resulting from seasonality, which produces a significant autocorrelation at a lag of 1 y.
(7 KB EPS)

Figure S2 Wavelet Coherence and Phase for the Square-Root-Transformed Incidence of CL and MEI For technical details see caption of Figure 3.
(2.3 MB PDF)

Figure S3 Mean Squared Error of the Forecasts Blue is for predictions with only 9 y of training data, and black, for all previous months preceding the prediction interval.
(8 KB EPS)

Protocol S1 Techniques to Study Nonstationary Time Series (51 KB DOC)

## Abbreviations

CL: cutaneous leishmaniasis
ENSO: El Niño Southern Oscillation
MEI: Multivariate ENSO Index
SST 4: Sea Surface Temperature 4

## References

[pmed-0030295-b001] Gratz NG (1999). Emerging and resurging vector borne diseases. Annu Rev Entomol.

[pmed-0030295-b002] Lainson R, Shaw JJ (1978). Epidemiology and ecology of leishmaniasis in Latin-America. Nature.

[pmed-0030295-b003] Chaves LF, Hernandez MJ (2004). Mathematical modelling of American cutaneous leishmaniasis: Incidental hosts and threshold conditions for infection persistence. Acta Trop.

[pmed-0030295-b004] Silveira FT, Lainson R, Corbett CE (2004). Clinical and immunopathological spectrum of American cutaneous leishmaniasis with special reference to the disease in Amazonian Brazil—A review. Mem Inst Oswaldo Cruz.

[pmed-0030295-b005] Zeledon R (1991). Cutaneous leishmaniais and Leishmania infantum. Trans R Soc Trop Med Hyg.

[pmed-0030295-b006] Ashford RW (1997). The leishmaniases as model zoonoses. Ann Trop Med Parasitol.

[pmed-0030295-b007] Scorza JV, Gomez I, McLure MT, Ramirez M (1968). Sobre las condiciones microclimaticas prevalentes en los microhabitats de flebotomos. Acta Biol Venez.

[pmed-0030295-b008] Marquez JC, Scorza JV (1984). Dinamica poblacional de Lutzomyia townsendi (Ortiz, 1959) (Diptera: Psychodidae) y su paridad en Trujillo, Venezuela. Bol Dir Malariol San Amb.

[pmed-0030295-b009] Feliciangeli MD, Rabinovich J (1998). Abundance of Lutzomyia ovallesi but no *Lu*. *gomezi* (Diptera: Psychodidae) correlated with cutaneous leishmaniasis incidence in north-central Venezuela. Med Vet Entomol.

[pmed-0030295-b010] Salomon OD, Wilson ML, Munstermann LE, Travi BL (2004). Spatial and temporal patterns of phlebotomine sand flies (Diptera: Psychodidae) in a cutaneous leishmaniasis focus in northern Argentina. J Med Entomol.

[pmed-0030295-b011] Rabinovich JE, Feliciangeli MD (2004). Parameters of Leishmania braziliensis transmission by indoor Lutzomyia ovallesi in Venezuela. Am J Trop Med Hyg.

[pmed-0030295-b012] Marrano NN, Mata LJ, Durack DT (1989). Cutaneous leishmaniasis in rural Costa Rica. Trans R Soc Trop Med Hyg.

[pmed-0030295-b013] Añez N, Nieves E, Cazorla D, Oviedo M, De Yarbuh AL (1994). Epidemiology of cutaneous leishmaniasis in Merida, Venezuela. III. Altitudinal distribution, age structure, natural infection and feeding behavior of sandflies and their relation to the risk of transmission. Ann Trop Med Parasitol.

[pmed-0030295-b014] Franke CR, Ziller M, Staubach C, Latif M (2002). Impact of the El Niño/Southern Oscillation on visceral leishmaniasis, Brazil. Emerg Infect Dis.

[pmed-0030295-b015] New M, Lister D, Hulme M, Makin I (2002). A high-resolution data set of surface climate over global land areas. Clim Res.

[pmed-0030295-b016] Wolter K, Timlin MS (1998). Measuring the strength of ENSO—How does 1997/98 rank?. Weather.

[pmed-0030295-b017] Venables WN, Ripley BD (2002). Modern applied statistics with S.

[pmed-0030295-b018] Gençay R, Selçuk F, Whitcher B (2002). An introduction to wavelets and other filtering methods in finance and economics.

[pmed-0030295-b019] Shumway RH, Stoffer DS (2000). Time series analysis and its applications.

[pmed-0030295-b020] Ghil M, Allen RM, Dettinger DM, Ide K, Kondrashov D (2002). Advanced spectral methods for climatic time series. Rev Geophys.

[pmed-0030295-b021] Maraun D, Kurths J (2004). Cross wavelet analysis: Significance testing and pitfalls. Nonlinear Proc Geophys.

[pmed-0030295-b022] Torrence C, Compo G (1998). A practical guide to wavelet analysis. Bull Am Meteor Soc.

[pmed-0030295-b023] Brockwell PJ, Davis RA (2002). Introduction to time series and forecasting, 2nd ed.

[pmed-0030295-b024] Durbin J, Koopman SJ (2001). Time series analysis by state space methods.

[pmed-0030295-b025] Ellner SP, Bailey BA, Bobashev GV, Gallant AR, Grenfell BT (1998). Noise and nonlinearity in measles epidemics: Combining mechanistic and statistical approaches to population modeling. Am Nat.

[pmed-0030295-b026] Patz JA, Hulme M, Rosenzweig C, Mitchell TD, Goldberg RA (2002). Regional warming and malaria resurgence. Nature.

[pmed-0030295-b027] Cazelles B, Chavez M, McMichael AJ, Hales S (2005). Nonstationary influence of El Niño on the synchronous dengue epidemics in Thailand. PLoS Med.

[pmed-0030295-b028] Abeku TA, De Vlas SJ, Boorsboom GJJM, Tadege A, Gebreyesus Y (2004). Effects of meteorological factors on epidemic malaria in Ethiopia: A statistical modelling approach based on theoretical reasoning. Parasitology.

[pmed-0030295-b029] Teklehaimanot HD, Schwartz J, Teklehaimanot A, Lipsitch M (2004). Weather-based prediction of Plasmodium falciparum malaria in epidemic-prone regions of Ethiopia II. Weather based prediction systems perform comparably to early detection systems in identifying times for interventions. Malar J.

[pmed-0030295-b030] Zhou G, Minakawa N, Githeko AK, Yan G (2004). Association between climate variability and malaria epidemics in the east African highlands. Proc Natl Acad Sci U S A.

[pmed-0030295-b031] Thomson MC, Mason SJ, Phindela T, Connor SJ (2005). Use of rainfall and sea surface temperature monitoring for malaria early warning in Botswana. Am J Trop Med Hyg.

[pmed-0030295-b032] Stenseth NC, Ottersen G, Hurrell JW, Mysterud A, Lima M (2003). Studying climate effects on ecology through the use of climate indices: The North Atlantic Oscillation, El Niño Southern Oscillation and beyond. Proc Biol Sci.

[pmed-0030295-b033] Faraway JJ (2005). Linear models with R.

[pmed-0030295-b034] Hallet TB, Coulson T, Pilkington JG, Clutton-Brock TH, Pemberton JM (2004). Why large-scale climate indices seem to predict ecological processes better than local weather. Nature.

[pmed-0030295-b035] Collier M, Webb RH (2002). Floods, droughts and climate change.

[pmed-0030295-b036] Bouma MJ (2003). Methodological problems and amendments to demonstrate effects of temperature on the epidemiology of malaria. A new perspective on the highland epidemics in Madagascar, 1972–1989. Trans R Soc Trop Med Hyg.

[pmed-0030295-b037] Leaney AJ (1977). Effect of temperature on *Leishmania* in sand flies. Parasitology.

[pmed-0030295-b038] Levins R, Epstein PR, Wilson ME, Morse SS, Slooff R (1993). Hantavirus disease emerging. Lancet.

[pmed-0030295-b039] Stone R (1993). The mouse-piñon connection. Science.

[pmed-0030295-b040] Levins R (1966). The strategy of model building in population biology. Am Sci.

