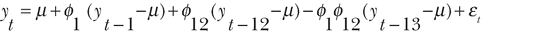# Correction: Climate Cycles and Forecasts of Cutaneous Leishmaniasis, a Nonstationary Vector-Borne Disease

**DOI:** 10.1371/journal.pmed.0040123

**Published:** 2007-03-27

**Authors:** Luis Fernando Chaves, Mercedes Pascual

In *PLoS Medicine*, volume 3, issue 8: doi:10.1371/journal.pmed.0030295

Equation 1 appeared incorrectly. It should read as:


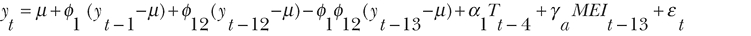


The equations in the legend of Table 1 were also incorrect. The full model should be:


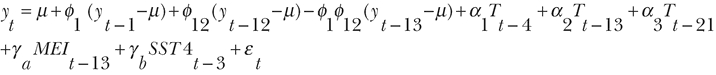


The null model should be: