# Supplementary material for: Climate Cycles and Forecasts of Cutaneous Leishmaniasis, a Nonstationary Vector-Borne Disease
Source: PLoS Med. 2006 Aug 15;3(8):e295. doi: 10.1371/journal.pmed.0030295 (PMC1539092; doi:10.1371/journal.pmed.0030295)
Supplement: Supplementary file 1 [file pmed.0030295.sg002.pdf]

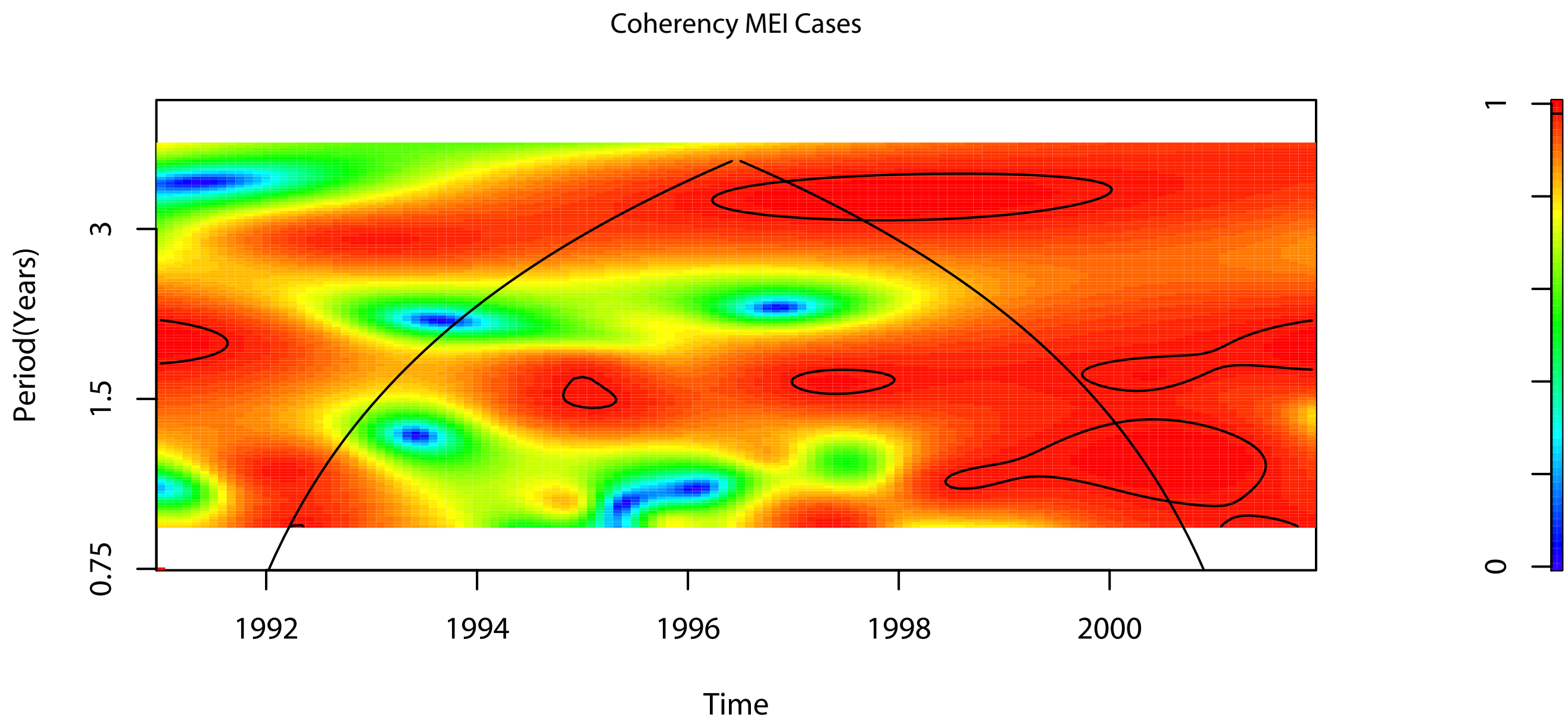
Coherency MEI Cases
Period(Years)
3
1.5
0.75
1992
1994
1996
1998
2000
Time
1
0

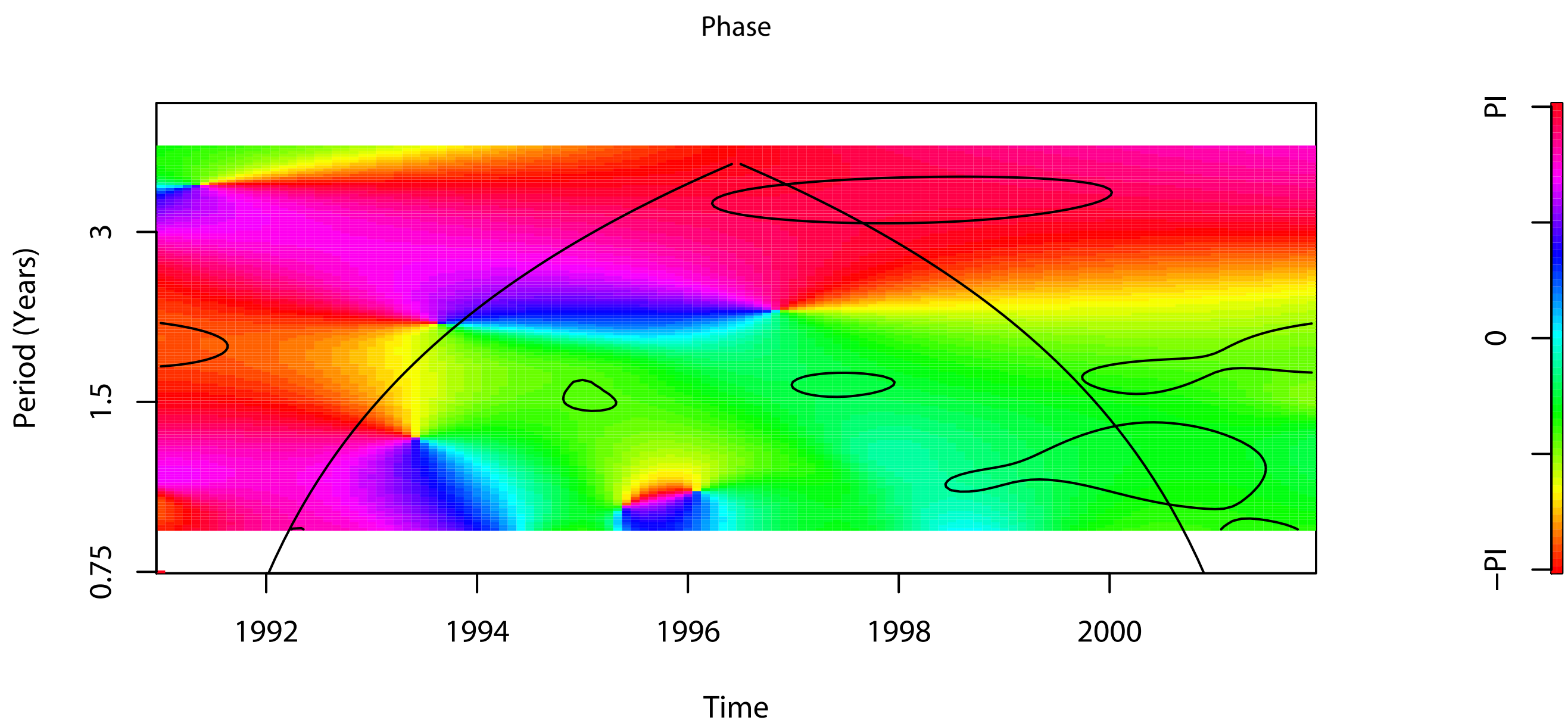
Phase
Period (Years)
3
1.5
0.75
1992
1994
1996
1998
2000
Time
PI
0
−PI
