# Supplementary material for: Climate Cycles and Forecasts of Cutaneous Leishmaniasis, a Nonstationary Vector-Borne Disease
Source: PLoS Med. 2006 Aug 15;3(8):e295. doi: 10.1371/journal.pmed.0030295 (PMC1539092; doi:10.1371/journal.pmed.0030295)
Supplement: Supplementary file 2 [file pmed.0030295.sd001.doc]

Protocol S1:

Most of the statistical procedures of this paper were implemented in R [s1]. For some specific procedures, additional software is indicated below.

**The periodogram and de-trending of the time series:**

The periodogram is defined as the squared spectral density of a time series [s2]:

$I(v_{k})=\left| n^{{-1}/2}\sum_{i=1}^{n} y_{t}e^{-2\pi\text{iv}_{k}t} \right|^{2}$	(a1)

The periodogram assumes that the series under study is stationary. One way to achieve stationarity is to detrend the time series. Here, we used the method of Discrete wavelet shrinkage. This method is based on the discrete wavelet transform (DWT) which in turn is a data transformation whose algorithm computes the wavelet coefficients of a series using an orthonormal and compactly supported function [s3]. As a result, the method can capture gross features of a series while focusing on finer details when necessary [s4, s5]. The advantage of DWT over other methods such as non-parametric splines or local polynomials, is this emphasis on the localized, as opposed to the global, behavior of the series [s4, s5]. The coefficients of the discrete wavelet transform are set (“shrunk”) to zero if they are smaller than a critical value, determined by the time series length and the variability of the wavelet coefficients for a given scale [s4, s5]. In this study, the Daubechies wavelet basis was used, symmetric and periodic edges were tested, the series was padded with its mean to ensure a dyadic (power of two) length, and the wavelet filter number was chosen using the basis that produces the median MSE to guarantee that the data is neither overfitted nor underfitted. Wavelets were fitted using the package wavethresh for R.

**Maximum entropy spectral density and non-Parametric de-noising of non- stationary time series**

We obtained the dominant frequency of the cycles with a second method, maximum entropy spectral density, to examine the robustness of our findings. The maximum entropy spectral density *Y*(*v_k_*) is applied to identify cycles in time series whose non-stationarity can be approximated by an autoregressive process [s6]. It is computed as follows:

$Y(v_{k})={\sigma_{w}^{2}}/{\left| 1+\sum_{j=1}^{p} \varphi_{j}e^{-2\pi\text{ijv}_{k}} \right|^{2}}$	(a2)

Again, peaks in this density indicate dominant frequencies. To successfully apply this technique, it is necessary to first separate signal from noise in the data, which we achieved with the two following methods:

1) Smoothing splines. The principle of this method is the minimization of a function that accounts for the trade-off between the fit, measured through the MSE, and λ, the degree of smoothness [s7]:

$\begin{aligned} y_{t}=\mu(x_{t})+\varepsilon_{t} \\ \sum\rho_{t}\left( y_{t}-\hat{\mu}(x_{t}) \right)^{2}+\lambda\int\left[ \mu\text{''}(x_{t}) \right]^{2} \end{aligned}$	(a3)

where ρ are weights used for robustness to outliers. The parameter λ was selected using generalized cross-validation [s7].

2) Singular spectrum analysis. This non-parametric technique separates trends and oscillatory components from noise in a time series. The method consists in the computation of the eigenvalues and eigenvectors from a covariance matrix {M} whose element m_ij_ is the covariance between lags i and j. The projection of the time series on the eigenvectors (the principal components of the matrix) reconstructs the pattern of variability associated with the selected eigenvalue, resulting in a de-noised time series [s6]. The eigenvalues themselves indicate how much variance is accounted for by the different components. For the SSA the toolkit described in [s6] was used.

# Wavelet Power Spectrum

The wavelet power spectrum (WPS_y_) is obtained from the so-called wavelet transform (WT_y_(s)). The best way to understand this transform is to consider that it is the product of the data and a function known as a wavelet (*ψ*) which is different from zero only for a range of values centered around the origin. By systematically translating the wavelet in time and computing this product, we obtain the transform

$\begin{aligned} \\ \text{WT}_{y}(s)=\sum_{j=1}^{n} y_{j}\psi*\left[ (j-i\delta t)/s \right] \end{aligned}$	(a4)

as a function of temporal scale s [s8, s9]. By contrast, the periodogram relies on the Fourier transform which uses sinusoidal functions that repeat continuously in time. This difference underlies the localization in time of the wavelet power spectrum given by the squared wavelet transform

$\begin{aligned} \text{WPS}_{y}=\left| \text{WT}_{y} \right|^{2} \\ \end{aligned}$	(a5)

# Wavelet Cross-Spectrum and patterns of association in non-stationary time series

Wavelet coherency is computed using the wavelet power spectrum of the two series under study as

$\text{WCO}_{\text{yx}}=$	(a6)

and varies between 0 and 1, with a value of 1 indicating maximum coherency. The time lag separating the two time series under study can also be determined by computing the phase of the cross wavelet spectrum, defined as the angle separating the real and imaginary parts of the wavelet cross spectrum [s8, s9]:

$\theta_{\text{yx}}=\text{tan}^{-1}\frac{\Im\left( \text{WT}_{y}\text{WT}_{x} \right)}{\Re\left( \text{WT}_{y}\text{WT}_{x} \right)}$	(a7)

Linear models and forecasts

One strategy for fitting time series models is to use their state space representation, that is, to find the underlying (not observed) process that produces the observed patterns in the time series [s2, s10].

State Space Representations

Time series can be seen as realizations of an unobserved stochastic process. Any unobserved process {y_t_} that can be expressed using observation and state equations has a state space representation [s2]. For the model process {y_t_} presented in (1) the state space representation can be obtained by introducing the following state vector:

$x_{t}=\left[ \begin{matrix} y_{t-\text{13}} \\ y_{t-\text{12}} \\ \vdots\\ y_{t-1} \\ y_{t} \end{matrix} \right]$	.	(a8)

The observation equation is:

$y_{t}=\left[ \begin{matrix} 0 & 0 & \cdots& 0 & 1 \end{matrix} \right]x_{t}$	(a9)

while the state equation is given by:

$x_{t+1}=\left[ \begin{matrix} 0 \\ 0 \\ \vdots\\ 0 \\ \mu\end{matrix} \right]+\left[ \begin{matrix} 0 & 1 & 0 & 0 & \cdots& 0 \\ 0 & 0 & 1 & \ddots& \cdots& 0 \\ \vdots& \vdots& \vdots& \ddots& \ddots& \vdots\\ 0 & 0 & 0 & \cdots& 0 & 1 \\ \varphi_{1}\varphi_{\text{12}} & \varphi_{\text{12}} & 0 & \cdots& 0 & \varphi_{1} \end{matrix} \right]x_{t}+\left[ \begin{matrix} 0 \\ 0 \\ \vdots\\ 0 \\ \alpha_{1} \end{matrix} \right]T_{t-4}+\left[ \begin{matrix} 0 \\ 0 \\ \vdots\\ 0 \\ \gamma_{a} \end{matrix} \right]\text{MEI}_{t-\text{13}}+\left[ \begin{matrix} 0 \\ 0 \\ \vdots\\ 0 \\ 1 \end{matrix} \right]\varepsilon_{t}$	(a10)

where the error is identically and normally distributed, i.e., $\varepsilon_{t}\text{\textasciitilde}N(0,\sigma_{w}^{2})$. For this unobserved process to have the same characteristics than those of the observed time series, it is necessary to address: (i) the correlation of the data over time (the smoothing problem), (ii) the fitting of every observation (the filtering problem) and (iii) the forecasting of future events (the prediction problem). These problems can be solved using Kalman Recursions [s2, s10]. The exact likelihood is computed via a state-space representation of the SAR process, and the innovations (i.e., residuals) and their variance found by a Kalman filter [s10].

**References**

s1 R Development Core Team (2005). R: A language and environment for statistical computing. R Foundation for Statistical Computing, Vienna, Austria. ISBN 3-900051-07-0, URL [http://www.R-project.org](http://www.R-project.org/).

s2 Brockwell PJ, Davis RA (2002) Introduction to Time Series and Forecasting. 2^nd^ ed. New York: Springer.

s3 Gençay R, Selçuk F, Whitcher B (2002) An introduction to wavelets and other filtering methods in finance and economics. San Diego: Academic Press.

s4 Shumway RH, Stoffer DS (2000) Time series analysis and its applications. New York: Springer. 572 p.

s5 Faraway JJ (2006) Extending the linear model with R: Generalized Linear, Mixed Effects and Non-parametric regression models. Boca Raton: Chapman and Hall CRC press.

s6 Ghil M, Allen RM, Dettinger DM, Ide K, Kondrashov D, Mann ME, Robertson A, Saunders A, Tian Y, Varadi F, Yiou P. (2002) Advanced spectral methods for climatic time series. Rev Geophys 40: 1-41

s7 Venables WN, Ripley BD (2002) Modern applied statistics with S. New York: Springer. 495 p.

s8 Maraun D Kurths J (2004) Cross wavelet analysis: significance testing and pitfalls. Nonlinear Proc Geoph. 11: 505-514

s9 Torrence C, Compo G (1998) A practical guide to wavelet analysis. Bull Am Meteor Soc 79: 61-78.

s10 Durbin J, Koopman SJ (2001) Time Series Analysis by State Space Methods. Oxford: Oxford University Press.
